# A text-mining based analysis of 100,000 tumours affecting dogs and cats in the United Kingdom

**DOI:** 10.1038/s41597-021-01039-x

**Published:** 2021-10-15

**Authors:** José Rodríguez, David R. Killick, Lorenzo Ressel, Antonio Espinosa de los Monteros, Angelo Santana, Samuel Beck, Francesco Cian, Jenny S. McKay, P. J. Noble, Gina L. Pinchbeck, David A. Singleton, Alan D. Radford

**Affiliations:** 1 Institute for Animal Health and Food Safety, University of Las Palmas de Gran Canaria, Canary Islands, Spain; 2 Institute of Infection, Veterinary and Ecological Sciences, University of Liverpool, Leahurst Campus, Chester High Road, Neston, CH64 7TE UK; 3 Mathematics Department, University of Las Palmas de Gran Canaria, Canary Islands, Spain; 4 VPG Histology (formerly Bridge), Horner Court, 637 Gloucester Road, Horfield, Bristol, BS7 0BJ UK; 5 Batt Laboratories, Venture Centre, Sir William Lyons Road, CV4 7EZ Coventry, United Kingdom; 6 IDEXX Laboratories Ltd., Grange House, Sandbeck Way, S22 7DN Wetherby, UK

**Keywords:** Epidemiology, Cancer epidemiology, Cancer epidemiology

## Abstract

Cancer is a major reason for veterinary consultation, especially in companion animals. Cancer surveillance plays a key role in prevention but opportunities for such surveillance in companion animals are limited by the lack of suitable veterinary population health infrastructures. In this paper we describe a pathology-based animal tumour registry (PTR) developed within the Small Animal Veterinary Surveillance Network (SAVSNET) built from electronic pathology records (EPR) submitted to this network. From an original collection of 180232 free text (non-structured) EPRs reported between April 2018 and June 2019, we used specific text-mining methodologies to identify 109895 neoplasias. These data were normalized to describe both the tumour (type and location) and the animal (breed, neutering status and veterinary practice postcode). The resulting PTR, the largest of its kind for companion animals to date, is an important research resource being able to facilitate a wide array of research in areas including surveillance, clinical decision making and comparative cancer biology.

## Abstract (summary)

**Table:** 

Measurement(s)	tumour
Technology Type(s)	digital curation • text mining
Factor Type(s)	animal species • breed • sex • neuter status
Sample Characteristic - Organism	Canis lupus familiaris • Felis catus
Sample Characteristic - Location	United Kingdom

Machine-accessible metadata file describing the reported data: 10.6084/m9.figshare.15074079

## Background & Summary

A tumour registry (TR) systematically collects and stores data allowing analysis and interpretation of these data from subjects with cancer providing useful information that may be used in different areas such as epidemiology, health care planning and monitoring^1^.

Based on the sources from which the information is collected, TRs can be hospital-based (HTR), pathology-based (PTR) or population-based^2^ with the latter being the gold standard in human oncology since it provides an unbiased profile of the cancer epidemiology in a defined population.

However, in the veterinary field, most previous animal TRs have been hospital-based or pathology-based^3^ neither of which are appropriate for cancer surveillance purposes by themselves given that both provide an incomplete (underreporting) and inaccurate (biased) sample based either on patient attendance at a given hospital or on laboratory-based surveillance.

Additionally, the lack of a background population to which compare the sample population affected by a tumour has remained a key limitation to developing population-based veterinary cancer registries^3^.

Researchers have tried to minimize this underreporting issue with different approaches to encourage participation of veterinary surgeons when it comes to submit samples for pathology diagnosis.

One approach adopted in TRs in the US (in 1968^4^ and 1978^5^), involved researchers asking all veterinarians in their respective areas to submit reports for all confirmed tumours. In an adaptation of this method in Italy, national^6^ and regional^7,8^ TRs have offered free histopathologic diagnosis for practitioners operating in their respective areas. A similar process was used in the “Cancer in the Dog” project (1990–1998)^9^, in Norway, and further updated in the Danish Veterinary Cancer Registry (2005–2008)^10^, in which veterinarians were invited to submit their tumour diagnosis (TD) through a web-based application. Veterinary insurance databases have also been used^11,12^ to obtain data from insured animal populations and finally, more recently, researchers have sought to harness data available in individual electronic pathology records (EPRs). In 2015, records from three diagnostic laboratories in Switzerland were used to create the Swiss Canine^13^ and Feline^14^ Cancer Registries, with more than 85000 tumour cases; the largest PTR so far.

Overall, animal TRs have been sporadic and usually been of limited duration^15^ and have never provided a comprehensive and detailed tumour dataset but a selection of their general results such as the most frequent tumours, locations, breed, age, etc.

Ideally, to create a useful surveillance tool, underlying data flows should be continuous and large enough to represent the population being studied. The data should be available in databases as near to real time as possible and be easily searchable without a requirement for particular technical skills. Here we describe our approach to meet these targets, of a sustainable PTR covering a large population with national coverage and open access, using a health informatic approach to efficiently extract anonymised tumour data from large volumes of routinely collected companion animal EPRs.

**Fig. 1:**
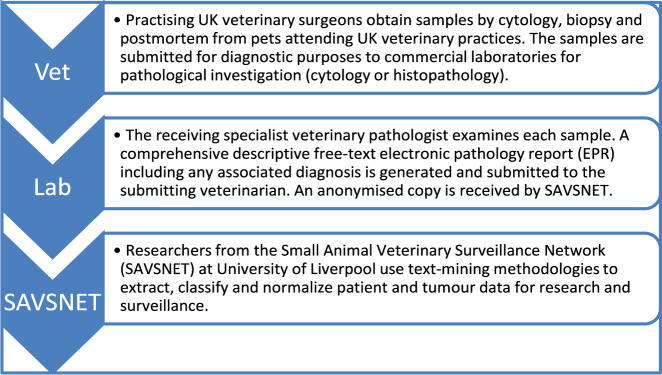
Schematic overview of the methodology.

Figure 1 shows our new approach that capitalises on existing data flows to an established national surveillance network (SAVSNET) which collects approximately 10000 diagnostic test results daily^16^ from participating laboratories, including haematology, pathology, biochemistry and infectious disease assays and uses them to support national surveillance and research^17,18^. For this study, we employed a text-mining methodology to extract, classify and normalize animal tumour data from three diagnostic laboratories, encompassing a total of 180232 canine and feline EPRs across the UK between April 2018 and June 2019. The result is a normalized animal PTR of 109895 tumours pertaining predominantly to dogs (91.6%) and diagnosed more commonly by histology (63.4%) than cytology (36.6%). The most common tumours in dogs were lipomas (21.7%), mast cell tumours (13.1%), and histiocytomas (7.7%) and in cats, lymphomas (14%) and squamous cell carcinomas (11.1%).

To our knowledge, this is the largest and most comprehensive animal PTR at a national level providing a reliable tool for veterinary practitioners and researchers as well as a baseline from which further studies can be developed although being always aware of the aforementioned limitations of PRTs to perform surveillance strategies.

Given the importance of companion animals as sentinels and models of human health, this registry and its future developments could play a significant role in comparative studies with human cancer registries under a ‘One Health’ approach.

## Methods

### Sample collection and preparation

This project used anonymized diagnostic test results submitted to the Small Animal Veterinary Surveillance Network (SAVSNET) at University of Liverpool between April 2018 and June 2019 by three UK diagnostic laboratories (IDEXX Laboratories, the Veterinary Pathology Group (VPG) and Batt Laboratories Ltd). During the study period and based on matching of postcodes, this included data from 2196 (48%) of the 4573 UK small animal veterinary practices in the Royal College of Veterinary Surgeons practice database (as used in former publications^17^), and from 120 of 121 UK postcode areas (only missing Hebrides), as well as Jersey, Guernsey and the Isle of Man. Each test result includes assay codes, test methodologies, sample descriptors, results (e.g. pathologist microscopic description) and pathologist interpretation as well as patient details including species, age, sex and a geographical locator based on the UK postcode of the submitting veterinary practice.

For this study, assay codes for cytology and histopathology were used to extract relevant animal and test data for manipulation in Microsoft Excel. Additionally, data were filtered by species to only include EPRs from cats and dogs.

In most cases, each row represented a unique laboratory submission, with columns containing information about the animal (such as breed, sex, neuter status), the sample taken (unique reference, date of record, assay type and postcode of the veterinary practitioner) and a free text description of the pathology report including diagnosis, prognosis, clinical summary, histology and comments. From some laboratories, data for individual samples (same sample reference) were supplied in a series of consecutive rows that required prior concatenation based on the sample reference number. Table 1 shows an example of a submission. For ease of manipulation, animal and lesion data were separated into two tables linked by the unique laboratory number.

**Table 1:** Example of a typical electronic pathology report used in this study.

	A	B	C	D	E	F	G	H
**1**	**LABNO**	**RECD**	**SPECIES**	**BREED**	**GENDER**	**PRACTICE_ID**	**ASSAY_CODE**	**RESCOMMENT1 (Pathology report)**
**2**	R.123	09/05/18	Canine	Labrador retriever	Female entire	XXXX XXX	HISTO	<br>DIAGNOSIS<br>1. Malignant mixed mammary gland tumour, gland three<br>2. Simple intratubular tubulopapillary carcinoma of the mammary gland, grade 2 - gland four<br>3.Consistent with MCT (second grade), forelimb<br>4. Low-grade cutaneous Lymphoma, highly likely><br>PROGNOSIS&nbsp;&nbsp; <br> Cautious <br> CLINICAL HISTORY <br> Two mammary masses and a forelimb mass removed. Samples from skin lesions were also taken <br><br> HISTOLOGY&nbsp;&nbsp; <br> Four specimens are submitted and evaluated…

### Data extraction

Data extraction from the free text pathology report (column H, Table 1), was carried out in three steps as described below.

#### STEP 1: Diagnosis and lesion location

Key words were used to extract specific sections of the text related to diagnosis and location of reported lesions. These key words could be slightly different depending on the laboratory from which the results emanated or the assay type (histology or cytology). For instance, in a histology report, the diagnosis appeared after the word DIAGNOSIS, while on a cytology report it was written after the words CYTOLOGICAL INTERPRETATION.

In order to facilitate explanation of the extraction process, an example of pre-extraction data is shown in Table 1. Data concerning tumour diagnosis was located between the words ‘DIAGNOSIS’ and either ‘PROGNOSIS’ or ‘CLINICAL HISTORY’ (since sometimes, a prognosis section was not included). Data pertaining to lesion location (LL) was reported normally either close to the TD or between CLINICAL HISTORY and HISTOLOGY, as can be seen in Table 1. Less frequently LL was positioned between HISTOLOGY and COMMENTS.

Given that LL could be written in one of these three different sections of the EPR, we developed a search which looked for LL in each of the possible positions within it. A prioritization system was then established selecting LL positioned between DIAGNOSIS and CLINICAL DIAGNOSIS over a LL between CLINICAL HISTORY and HISTOLOGY which itself was prioritised over an LL written in the histology section between HISTOLOGY and COMMENTS.

The positions of each of these key words (DIAGNOSIS, PROGNOSIS, CLINICAL HISTORY, and HISTOLOGY) were identified using the Excel function SEARCH (columns C-F, Table 2). Subsequently, the MID function was used to extract the text potentially containing TD (between DIAGNOSIS and PROGNOSIS) and LL (as explained above) into separate columns (columns G and H, Table 2).

**Table 2:** Data extraction.

	A	B	C	D	E	F	G	H
**1**	**LABNO**	**RESCOMMENT1 (Pathology report)**	**DIAGNOSIS**	**PROGNOSIS**	**CLINICAL HISTORY**	**HISTOLOGY**	**DIAGNOSIS INFO**	**LOCATION INFO**
**2**	R.123	<br>DIAGNOSIS <br>1. Malignant mixed mammary gland tumour, gland three <br>2. Simple intratubular tubulopapillary carcinoma of the mammary gland, grade 2 - gland four<br>3.Consistent with MCT (second grade), forelimb<br>4. Low-grade cutaneous Lymphoma, highly likely><br>PROGNOSIS&nbsp;&nbsp;<br>Cautious<br>CLINICAL HISTORY<br>Two mammary masses and a forelimb mass removed. Samples from skin lesions were also taken<br><br>HISTOLOGY&nbsp;&nbsp;<br>Four specimens are submitted and evaluated…	5	271	309	427	DIAGNOSIS<br>1. Malignant mixed mammary gland tumour, gland three<br>2. Simple intratubular tubulopapillary carcinoma of the mammary gland, grade 2 - gland four<br>3.Consistent with MCT (second grade), forelimb<br>4. Low-grade cutaneous Lymphoma, highly likely><br>	CLINICAL HISTORY<br>Two mammary masses and a forelimb mass removed. Samples from skin lesions were also taken<br><br>

Step 1: Tumour diagnosis (TD) and lesion location (LL) information were automatically extracted from the pathology free text based on key words used to delimit sections of the report.

#### STEP 2: Separation into single lesions

In some cases, multiple lesions were recorded in a single submission so we decided to look for a maximum of six possible tumours in each animal since the frequency of report numbers repeated up to six times was small (approximately 1% of all the reports).

The vast majority of such cases were identified as an individual diagnosis preceded by a number or a letter, as a delimiter (as shown in column G, Table 3 for an animal suffering from four tumours). Data relating to each of these lesions was extracted using the SEARCH function to locate the separating characters (“1.” to “6.” or “A” to “F”) and the MID function to extract the data pertaining to the individual lesion into a separate column (column M-P, Table 3).

**Table 3:** Separation of single lesions in a case with four different tumours.

	G	H	I	J	K	L	M	N	O	P
**1**	**DIAGNOSIS INFO**	**LOCATION INFO**	1.	2.	3.	4.	1^st^ tumour	2^nd^ tumour	3^rd^ tumour	4^th^ tumour
**2**	DIAGNOSIS<br>1. Malignant mixed mammary gland tumour, gland three<br>2. Simple intratubular tubulopapillary carcinoma of the mammary gland, grade 2 - gland four<br>3.Consistent with MCT (second grade), forelimb<br>4. Low-grade cutaneous Lymphoma, highly likely><br>	CLINICAL HISTORY<br>Two mammary masses and a forelimb mass removed. Samples from skin lesions were also taken<br> <br>	14	70	165	215	1. Malignant mixed mammary gland tumour, gland three<br>	2. Simple intratubular tubulopapillary carcinoma of the mammary gland, grade 2 - gland four<br>	3.Consistent with MCT (second grade), forelimb<br>	4. Low-grade cutaneous Lymphoma, highly likely><br>

#### STEP 3: Identification of single lesions and tumour classifiers

We next identified tumour types, locations and grades recorded within the now separated lesion free text. First, all unique lesional free texts from columns M to P in Table 3 were copied into a single column of a new spreadsheet (e.g Table 4 column B).

**Table 4:** A new column with all the individual lesions.

	A	B	C	D	E	F	G	H
**1**	**Tumour_ref**	**Lesion description**	**Primary_tumour**	**Grade_2_tier (Kiupel for MCT).**	**Grade_3_tier (Patnaik for MCT).**	**Differentiation**	**Uncertain terms**	**Location**
**2**	R.123-T.1	1. Malignant mixed mammary gland tumour, gland three<br>	Mixed mammary gland tumour			Malignant		Mammary gland
**3**	R.123-T.2	2. Simple intratubular tubulopapillary carcinoma of the mammary gland, second grade - gland four<br>	Simple intratubular tubulopapillary carcinoma		grade 2			Mammary gland
**4**	R.123-T.3	3.Consistent with MCT (second grade), forelimb<br>	MCT		second grade		Consistent with	Forelimb
**5**	R.123-T.4	4. Low-grade cutaneous Lymphoma, highly likely><br>	Lymphoma	Low-grade			Highly likely	Skin

As we planned to make the PTR search and sortable by both TD and tumour characteristics, we parsed the data into individual columns for each data item. An iterative process was then used in Table 4 to identify text relating to each TD (column C), tumour grade (columns D and E), degree of differentiation (column F), the location of the tumour (column H) and probability terms related to the pathologist’s confidence in the TD such as “highly likely”, “probable” or “consistent with” (column G).

This was accomplished using a nested array operation in Excel to identify text within all these above columns (C-H, Table 4) that matched a series of curated lists^19^ compiled iteratively as a series of six look up tables (columns A-F, Table 5).

**Table 5:** A sample of the six look up tables^19^ created to search for specific text in the pathology free text (*grades in the dataset have been kept for the tumours indicated in the Data records section, columns J and K. In this table they are shown just as an example).

	A	B	C	D	E	F
**1**	**Primary_tumour**	**Grade_2_tier**	**Grade_3_tier**	**Differentiation**	**Uncertain terms**	**Tumour_location**
**2**	**N = 1808**	**N = 14**	**N = 9**	**N = 22**	**N = 39**	**N = 398**
**3**	(hepatoid gland) adenocarcinoma	Low grade		Malignant transformation	Compatible with	Anal region
**4**	(hepatoid gland) adenoma			Benign	Consistent with	Anal sac
**5**	(hepatoid gland) carcinoma	Low-grade		Malignant	Favoured	Anal gland
**6**	(hepatoid, circumanal) gland adenocarcinoma	High-grade		Poorly differentiated	Follow	Perianal
**7**	(hepatoid, circumanal) gland adenoma			Moderately differentiated	Highly likely	Anal
**8**	(hepatoid, circumanal) gland carcinoma	Intermediate-grade		Well differentiated	Highly suggestive	Hindlimb
**9**	adenocarcinoma (anaplastic)			Poorly differentiated	Inconclusive	Forelimb
**10**	Adenocarcinoma arising in mixed gland mammary			Moderately-differentiated	Indicative of	Axilla
**11**	Adenocarcinoma arising in mixed mammary			Well-differentiated	Keeping with	Brain
**12**	adenocarcinoma of the anal sac apocrine glands			Undifferentiated	Likely	Chest
**13**	adenocarcinoma of the apocrine glands					
**14**	adenocarcinoma of the mammary gland, tubulopapillary		Grade II			Mammary gland
**15**	adenocarcinoma of the Parathyroid gland					
**16**	Apocrine ductal carcinoma					

In particular, the curated reference TD list (column A, Table 5) was created mainly from ‘Tumors in Domestic Animals’^20^ (a standard and comprehensive text in the field).

Each nested array formula took the following general format: = INDEX(**Primary_tumour**,MATCH(TRUE,ISNUMBER(SEARCH(Primary_tumour;$B2)),0)).

As an example, the above formula searches for text in a specific cell of Table 4, column B that matches any of the terms in Table 5, column A, starting from the top of this column. This function works downwards from the top of the column until it reaches a matching entry. Once a match is identified it is copied to a new cell (in this case Table 4, column C). Consequently, only a single match was recorded.

Each column of Table 5 was established iteratively using this approach. The reference tables were first populated with generic “capture terms” based on domain knowledge.

For example: Column A included words like tumour, carcinoma, neoplasia etc, whereas Column F included head, neck, mammary etc. Patterns found by these capture terms in the first search were checked and specific tumour names added back to the top of Table 5 column A as necessary. As an example of this, in a first search with only generic “capture terms” (such as “Tumour”, “Carcinoma” or “Neoplasia”) in column A Table 5, cells C2 and C3 of Table 4 would have shown the terms “Tumour” and “Carcinoma” instead of “Mixed mammary gland tumour” and “Simple intratubular tubulopapillary carcinoma” respectively. Eventually, however, once these more specific TD terms were added on the top of the general ones in column A Table 5, they were the ones assigned to the record (instead of the general ones) and shown in cells C2 and C3 of Table 4.

After each round of searching and augmenting the look up tables, 200 records from Table 4 that did not match on a specific column in Table 5 were read, and any newly identified terms added to Table 5. This process was repeated iteratively until no new terms were identified in 200 read texts.

Data entries possible in each column of Table 5 are as follows:

**COLUMN A - Type of primary tumour**: It includes 1808 general expressions of tumour types.

We used a case definition outlined in ‘Tumors of Domestic Animals’^20^ and former publications^12,13^ in such a way that those tumours considered specifically as neoplasms or tumours in these texts were included in the PTR while other lesions classified as hamartomas, cysts or tumour-like masses, were excluded.

**COLUMN B - Grade 2 tier (Kiupel for MCT)**: here we have included the terms low-grade, intermediate-grade and high-grade where recorded.

**COLUMN C- Grade 3 tier (Patnaik for MCT)**: here we have included the terms grade I, grade II and grade III where recorded.

**COLUMN D - Differentiation:** this list includes terms related to the differentiation of the tumour such as “Benign”, “Malignant”, “Undifferentiated” or “Well- differentiated”.

**COLUMN E - Uncertain terms:** this category contains terms that may be added to the TD when the pathologist has any doubt about the diagnosis such as “highly likely” lymphoma or “consistent with” lipoma.

**COLUMN F - Location:** In this category we have included anatomical terms related to the tumour location although some caution must be considered since sometimes this is not technically the tumour location but rather the location where a first lesion was detected in the animal and motivated the first visit to the vet.

### Data normalization

As a result of applying the aforementioned methodology, different ways of referring to the same kind of data were obtained, as exemplified by Table 6 Column A, where an adenoma of hepatoid glands has been referred to by the pathologists in six different ways. Similar problems were identified for LL (e.g. leg and limb), degree of differentiation (e.g. “grade 1” and “grade I”), as well as dog and cat breeds from the animal data spreadsheet (e.g. “Labrador Retriever” and “Retriever, Labrador”). These were mapped to’preferred’ terms^19^ using the VLOOKUP Excel function. The preferred terms were themselves either based on domain expertise, or for tumour types using the different tumour lists found in ‘Tumors of Domestic Animals’^19,20^. An alternative would have been to use WHO ICD-O terms, but these are not fully compatible with veterinary tumours at this time. Once a veterinary ICD-O has been finalised it would be relatively straightforward to code the PTR data to that format. Dog breeds were mapped to those recognised by the Fédération Cynologique Internationale (FCI) and the American Kennel Club (AKC) while cat breeds were mapped to those recognised by the Fédération Internationale Féline (FIFE) and the International Cat Association (TICA) augmented by recent additions based on popular hybrids (e.g. labradoodle).

**Table 6:** The same kind of tumour counted with different denominations.

	A	B	C
**1**	**Results from the tumour types**	**Number of times each term has been counted**	**Unique term for a certain tumour**.
**2**	(hepatoid gland) adenoma	5	Adenoma of the hepatoid glands
**3**	(hepatoid, circumanal) gland adenoma	5	
**4**	Perianal (hepatoid) adenoma	4	
**5**	Hepatoid adenoma	10	
**6**	Hepatoid gland adenoma	3	
**7**	Adenoma of the hepatoid glands	6	

Once both tumour and animal dataset were completely processed and normalised separately, they were merged using functions OPENXLSX and MERGE with RStudio software (RStudio Version 1.2.1335) by using a bespoke R script^19^.

The end result is a dataset with an easy to read structure as shown in Table 7 although additional details of the actual dataset are described in the Data Records section.

**Table 7:** Basic structure of the dataset after merging tumour and animal data.

Report Ref	Tumour Ref	Result Date	Species	Breed	Gender	Anomymous_PracticeID	Histo_Cyto	Tumours_in_the_report	Primary_tumour	Grade_2_tier (Kiupel for MCT).	Grade_3_tier (Patnaik for MCT).	Differentiation	Location	Uncertain terms
R.123	R.123-T.1	09/05/18	C*	LR*	FE*	XXXX XXX	H*	4	Mixed tumour			Malignant	MG*	
R.123	R.123-T.2	09/05/18	C*	LR*	FE*	XXXX XXX	H*	4	Simple tubulo-papillary carcinoma		2		MG*	
R.123	R.123-T.3	09/05/18	C*	LR*	FE*	XXXX XXX	H*	4	Mast cell tumour		2		Forelimb	Consistent with
R.123	R.123-T.4	09/05/18	C*	LR*	FE*	XXXX XXX	H*	4	Lymphoma	Low-grade			Skin	Highly likely

**Fig. 2:**
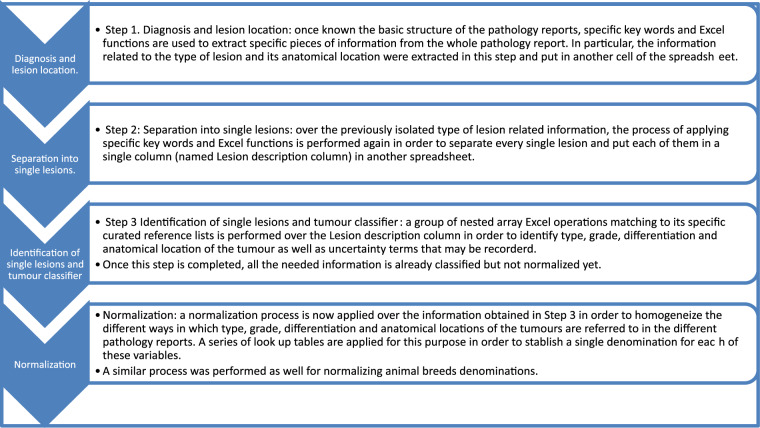
Schematic overview of Data extraction (three steps) and normalization processes.

Figure 2 shows an over-arching explanation of both the data extraction (three steps) and normalization processes. Additionally, for an easier understanding of the whole process, a spreadsheet containing a sample of reports with the formulas performing the aforementioned tasks is available online^19^.

## Data Records

The final SAVSNET PTR dataset^19^ consists of 109895 rows tumours and 15 columns (columns A to O) which are described below.

A. ReportRef: N = 93941 pathology reports (“R.” stands for report). It indicates the number of the pathology report (linked anonymised from the submitting laboratory report number). This value if repeated in different rows indicates those cases where reports contain multiple tumours.
  From the original 180232 pathology reports, 93941 reported at least one tumour while the other 86291 reports with no tumour were discarded.
B. TumourRef: N = 109895 tumour references within the 93941 pathology reports. It indicates the reference of the tumour, so for example tumours R.10000-T.1 and R.10000-T.2 means that there are two different tumours in report R.10000.
C. ResultDate: the date the tumour was reported by the lab.
D. Species: 180 canine breeds or 39 feline breeds.
E. Breed: breed of the dog (N = 180, top 5 unknown, Crossbreed, Labrador Retriever, Staffordshire Bull Terrier, Cocker Spaniel) or cat (N = 39, top 5 Domestic Short Hair, unknown, Domestic Long Hair, British Blue, Maine Coon).
F. Gender: gender of the cat or dog including neuter status where known. From a total of 93941 pathology reports, 85435 were from dogs and 8506 from cats. Within dogs, 41570 female, 41574 male and 2291 unknown. Within cats, 4275 females, 3969 males and 262 unknown.
G. Anonymous_PracticeID: indicates the practice where the sample was taken. During the study period and based on matching of postcodes (these have been anonymized since real postcodes cannot be published under SAVSNET´s ethical approval; more details are explained in the Usage notes section), this included data from 2196 (48%) of the 4573 UK small animal veterinary practices in the Royal College of Veterinary Surgeons practice database (as used in former publications^17^).
H. Histo_Cyto: indicates whether the tumour was analyzed by cytology (N = 40252) or histology (N = 69643).
I. Tumours_in_the_report: the number of tumours a report contains. 1 tumour = 82479, 2 = tumours 16904, 3 = tumours 6066, 4 = tumours 2480, 5 = tumours 1210, 6 tumours = 756. Median 1 for both cats and dogs.
J. Primary_tumour: indicates the specific name of the tumour (121 in total). Top 3 cat (Lymphoma, Squamous cell carcinoma, Carcinoma_others) and dog (Lipoma, Mast cell tumour, Histiocytoma).
K. Grade_2_tier (Kiupel for MCT): indicates the 2 tiers grade for lymphomas and Kiupel for mast cell tumours.
L. Grade_3_tier (Patnaik for MCT): indicates the 3 tiers grade for mammary carcinomas and soft tissue sarcomas and Patnaik for mast cell tumours.
M. Differentiation: provides additional information about the diagnosis 12 terms used in total. Most common: “malignant”, “benign”, “well differentiated”.
N. Location: indicates the tumour location on the patient. 88 locations in total. Top 3 cat (Mammary gland, Skin, Neck) and dog (Mammary gland, Skin, Thorax)
O. Uncertainty_terms: this category contains terms such as “highly likely” or “consistent with” that may be added to the TD when the pathologist has any doubt about the diagnosis. Most common: “Consistent with”, “Possible”, “Probable”.

The final dataset describes a PTR that includes a list of 121 different types of tumours that appear at least 10 times in the database. However, within this 121 TD list there are six non-specific terms (Carcinoma_others, Adenoma_others, Epithelioma_others,Epithelial tumour_others, Mesenchymal_neoplasias_others and Neoplasia_Tumours_others) which, in turn, either include other specific tumour types appearing less than 10 times such as for example some Leukaemias (included within the term Neoplasia_Tumours_others) or some Islet cell carcinomas (included within the term Carcinoma_others) as well as other tumours reported only using general terms such as “Mammary gland carcinoma” or “Rectal Adenoma” without additional information about the type or tumour it consisted. Additionally, some types of tumour such as multiple myelomas and plasmacytomas were aggregated under the term “Plasma cell tumour”. In all these cases, LL and differentiation may be particularly useful for indicating the tumour type. For example: from the 4838 “Epithelial tumour_others” found in the dataset, 42 are located in the liver. Further, one of them are said to be “Well differentiated” and one is said to be “Benign” further supporting the impression that they are both hepatocellular adenomas. Conversely, from these 1 of the 42 liver epithelial tumours, one is said to be “Malignant”; so this is more likely to be an hepatocellular carcinoma.

In regard to LL; this information is derived either from the histology or more commonly from a transposition of the lesion description on the submission form into the pathology report, and has certain limitations. Firstly, therefore, the location may indicate a region of the body rather than a precise anatomical location. Three examples are that several lipomas are said to be located in the mammary gland according to the dataset due to the fact that they are reported as “lipomas close to the mammary gland” or “Lipomas: mammary gland region”. Given that these reports use the term “mammary gland” to set a LL instead of using other words such as “thorax” or “abdomen” some of these tumours are recorded in the PTR as LL ‘mammary gland’ when in fact they may have been overlying the gland or just in that general location. Secondly, when there are multiple tumours without a clear separation between them and their respective locations, an erroneous LL may rarely appear as, for example, “a seminoma in the head”. Finally, the user should be aware that anatomical structure and LL are sometimes not differentiated in the report for the same reason, hence, a tumour affecting a limb could in principle be affecting any of the structures of the limb, although in practice it is often evident from the tumour type which the most likely structure is.

It must also be pointed out, concerning lipomas, that given the cells of these tumours are identical to those in normal adipose tissue, it is not possible to differentiate between lipoma and normal subcutaneous fat by cytology alone; this is a clinical decision. Consequently, readers are encouraged to check the Histo_Cyto column when it comes to considering such tumour type where the diagnostic procedure could impact the diagnostic accuracy.

## Technical Validation

### Checking for accuracy of our exploratory text-mining methodology in determining TD

The ultimate goal of this system is to automate the collation of groups of tumour types for further review (for example in epidemiological studies). With this in mind, we designed a technical validation to assess the accuracy of the text-mining procedure in identifying the correct TD from each EPR. To do this we compared the text mining results to a gold standard of expert opinion. Firstly, two experts, one a board-certified medical oncologist (DK) and the other a board-certified veterinary pathologist (LR), each reviewed a random sample of 200 unique EPRs with no overlap, recording their own TD; to avoid any possible bias, both reviewers were blinded to the results obtained by the text-mining procedure.

Secondly, the assessment of the 400 expertly reviewed rows was compared to the output from the text mining procedure by a third expert (AE), a Professor of Veterinary Pathology, who was also blinded to the origin of both groups of results in such a way that he was unaware which of the two results were from the expert, and which were derived by text mining.

Overall, for reports in which a single tumour was present (298 out of 400), 286 successful results were observed giving an accuracy of 96%. For the multiple tumour group (102 out of 400), the accuracy was 89% with 91 successful results observed.

However, when considered separately, cytology and histology reports showed some differences in accuracy.

In the single tumour group, which included 144 cytology and 154 histology reports, accuracy was 92% (133 successful results) and 99% (153 successful results) respectively.

In the multiple tumour group, which included 72 cytology and 30 histology reports, accuracy was 88% (63 successful results) and 93% (28 successful results) respectively. Table 8 provides a summary of the results obtained by the technical validation.

**Table 8:** A summary of the results obtained by the technical validation process.

TYPE OF REPORT (n = 400)	SINGLE TUMOUR GROUP (n = 298)			MULTIPLE TUMOUR GROUP (n = 102)		
	Total	Results matched	Accuracy by type of report.	Total	Results matched	Accuracy by type of report.
Cytology (n = 216)	144	133	92%	72	63	88%
Histology (n = 184)	154	153	99%	30	28	93%
Overall accuracy by group (single and multiple).	96%			89%		

Overall, there were 23 reports, shown in Table 9, where the diagnosis provided by the data mining was incorrect according to the experts. In this regard, five reasons for this misdiagnosis were identified:

**Table 9:** A list with the 23 misdiagnosed reports found in the technical validation. NT* = Non tumour, CI*: Cytological Interpretation.

Histo_Cyto	Diagnosis SAVSNET-PTR	Diagnosis Experts	Reason	Comment about misdiagnosis	Frequency (N = 23)
Cyto	NT*.	Lipoma.	1	The term “lipoma” was not written in the CI*.	5
Cyto	One lipoma.	Four lipomas.	2	No delimiters between the different tumours.	2
Histo	Four plasma cell tumours and a peripheral odontogenic fibroma.	Five plasma cell tumours and a peripheral odontogenic fibroma.	2	No delimiters between the different tumours.	1
Cyto	Lymphoma.	NT*.	3	Diagnosis mentions “Lymphoma not excluded”.	1
Cyto	Thyroid epithelial neoplasia.	Thyroid carcinoma.	5	Specific diagnosis not written in the CI*.	1
Cyto	Melanocytic tumour.	NT*.	3	Diagnosis mentions “cannot exclude a melanocytic neoplasm”.	1
Cyto	Lipoma.	Lipoma and Basal cell tumour.	2	No delimiters between the different tumours.	1
Cyto	One lipoma.	Tow lipomas.	2	No delimiters between the different tumours.	1
Cyto	One lipoma.	Three lipomas.	2	No delimiters between the different tumours.	1
Histo	One seminoma.	Two seminomas.	2	No delimiters between the different tumours.	1
Cyto	Neoplasia-Tumour_others.	Lipoma.	1	The term “lipoma” was not written in the CI*	1
Cyto	Hepatoid (perianal) carcinoma.	Anal sac carcinoma.	4	Wrong location.	1
Cyto	Epithelial tumour.	NT*.	3	Diagnosis mentions “Possible lymphoid or epithelial neoplasia”	1
Cyto	Lipoma in oral cavity.	NT*.	3	Diagnosis mentions “Consistent with aspiration of adipose tissue, lipoma highly likely”	1
Histo	Meibomian adenoma	Meibomian hyperplasia (NT*).	3	Diagnosis mentions “Early Meibomian adenoma” as a differential diagnosis.	1
Cyto	Mesenchymal neoplasia.	Soft tissue sarcoma.	5	Specific diagnosis not written in the CI*.	1
Cyto	Carcinoma	NT*.	3	Diagnosis mentions “Carcinomatosis effusion”.	1
Cyto	Epithelial tumour in abdomen.	Thyroid neoplasia.	4	Wrong location.	1

**Reason 1- Lipomas**. Reporting a lipoma was missed six times by text-mining because the original report did not include the word “lipoma” in the Cytological interpretation section but rather expressions such as “fat tissue aspiration” or “aspiration of lipid material”. In these cases, the experts determined that the most likely diagnosis was a lipoma based on information in other sections of the report including the clinical summary, the cytological description and the comments.

**Reason 2 - Missing tumours in reports with multiple tumours**. In seven cases, reports containing multiple tumours were partially misclassified by text mining because delimiters between tumours were not used the usual way. For example, instead of using numbers as delimiters (1. Seminoma, 2. Seminoma), the report may have quoted “Seminoma in both testicles” or “All four sites: Lipoma”. In these cases, the current text mining approach would only identify the first tumour type mentioned in the report.

**Reason 3 – Not detecting provisional diagnoses**. In six cases, an NT or inconclusive diagnosis were misclassified by text mining because the report included expressions such as “…cannot exclude a melanocytic neoplasm”, “Lymphoma not excluded” or “Meibomian gland hyperplasia (DDx early Meibomian gland adenoma)”. In these particular examples, a diagnosis of a Melanocytic tumour, a Lymphoma and a Meibomian adenoma respectively were given wrongly.

**Reason 4 – Wrong location**. Two reports were misdiagnosed because a wrong tumour location was pulled out. Firstly, a carcinoma in the perianal area (hepatoid carcinoma) was diagnosed by the data mining when the actual location were the anal sacs glands (apocrine glands), so the experts diagnosed an anal sac carcinoma instead of an hepatoid carcinoma. Secondly, in a multiple tumour report without a clear separation between the different lesions, a report containing an epithelial tumour in the thyroid gland and an inflammatory lesion in the abdomen was misdiagnosed as an epithelial tumour in the abdomen.

**Reason 5 – Incomplete diagnosis. Two reports were partially misdiagnosed because the complete diagnosis was not written in the report**. One report was given the diagnosis of an Epithelial tumour (without specifying if benign or malign) in the thyroid gland when the actual diagnosis was a Thyroid carcinoma. Equally, a diagnosis of a mesenchymal neoplasia was given when the correct diagnosis was a soft tissue sarcoma.

## Usage Notes

### Limitations and proper uses of the SAVSNET PTR

In spite of the large amount of information provided by the SAVSNET PTR and the wide geographic area (nationwide) from which these data are received, it should be pointed out that in this paper we are not providing any data or estimation about the reference population or population at risk which has been a key limitation to former TRs in the veterinary field over the last decades. As mentioned earlier, the SAVSNET PTR has received data from just three veterinary diagnostic labs so, consequently, we are not providing data on all the tumours diagnosed in the UK since not all veterinary diagnostic labs submit data to SAVSNET. Indeed, others have shown that tumour registries based on this kind of data suffer both from underreporting (not all diagnosed tumours in the area under study are submitted) and underascertainment (not all tumours detected in a clinical examination have samples submitted for diagnosis)^21^. Because of this, the data from this dataset cannot be extrapolated to the entire populations of dogs and cats in the UK due to the potential for systematic bias in the reporting and ascertainment.

In other words, this is not a population-based tumour registry but a pathology-based tumour registry and, therefore, this data should not be used to calculate tumour incidence rates in the whole population nor should it be considered as a reliable resource to obtain conclusions or estimations about risks related to any breed or tumour type within the whole UK populations of dogs and cats. For example, within the total 93,941 reports presented in this dataset, 10,095 came from Labrador Retriever dogs. However, this breed is also considered the most common in the UK population of vet visiting dogs^22^.

Clearly, in the absence of clear denominator, it cannot be inferred that Labrador Retrievers are the most at risk of cancer in the UK.

In this regard, the Small Animal Veterinary Surveillance Network is looking to produce population denominator surrogates using electronic health records of dogs and cats visiting first opinion veterinary practices and estimates of overall UK dog populations.

Taking these limitations into account, the information presented in the dataset could however provide descriptions of the proportional distribution of tumour types within breeds and\or different neuter status or sex among animals included in our dataset. Additionally, as others have done before in similar research projects^23^, it would be possible to perform simple statistical analysis to analyze the influence of the different variables (breed, sex, neuter status) on the appearance of the different tumours within the dataset although with the caution of being always aware that any result obtained from this analysis would be referred and limited to the animals within the dataset and not to the whole population.

The final dataset can be fully manipulated in Excel, using simple functions like pivot tables, thereby allowing the association between factors such as sex or breed and tumour types to be readily explored within the cohort of animals included in the dataset.

### Limitations from secondary data sources

The SAVSNET tumour registry relies on information provided by diagnostic labs. All the data related to sex, neuter status, breed, etc., should be considered secondary data showing a lot of diversity given the large amount contained in the dataset. For that reason, a normalization process was performed in the Methods section.

Readers should consequently consider that normalized secondary data may not be as accurate as primary data obtained directly from the researchers.

### Multiple counting of the same tumour and how to work with pathology reports instead of tumours

Given that this is a tumour diagnosis-based database, and no unique ID for animals is provided, it may be possible that individual dogs or cats might have more than one sample of the same tumour in the database (for example because owners wanted a second opinion and decided to take another sample of the same tumour in a different veterinary practitioner). This would lead to multiple counting of the same tumour, breed, etc.

In some cases, users may be interested in data related to the animals or regions presented in this dataset rather than in the tumours themselves and so, for this purpose, users can work at the level of 93941 pathology reports (n = 93941), rather than at the level of individual tumours (n = 109895).

### Raw data access

The histopathology reports on which the final published dataset is based cannot be made available in an open access format as they contain clinically and financially sensitive information relating to the diagnostic laboratory or veterinary practice, as well as rare references to animal names. However, access may be possible by reasonable request for use in line with SAVSNET´s overarching ethical approval from the University of Liverpool. Researchers wishing to access the raw data need to apply for access here https://www.liverpool.ac.uk/savsnet/using-savsnet-data-for-research/ where assessment will be made based on objectives, publication strategy and track record. In some cases, an access fee may be chargeable. Those successful in their application will need to complete a data user agreement^19^ which details the necessary safeguards for these data.

Under SAVSNET’s ethical approval, owner consent is not required as SAVSNET does not collect any data that could identify them. Postcodes of the submitting practice for each test performed are collected; under our ethical approval, these postcodes cannot be published. Instead, we have described in the text the percent of veterinary practices as an indicator of coverage provided in the existing PTR and provided an anonymised practice code for each sample in the PTR itself to allow researchers to explore clustering of tumours by practice.

## Data Availability

The bespoke R script can be accessed at SAVSNET TUMOR REGISTRY DOCUMENTS figshare collection^19^ with no restriction to access.
